# Supplementary material for: Are carbon emissions trading and green financial instruments synergistic? -Comprehensive quantitative research based on content analysis
Source: PLoS One. 2024 Mar 7;19(3):e0298601. doi: 10.1371/journal.pone.0298601 (PMC10919601; doi:10.1371/journal.pone.0298601)
Supplement: S1 Data — (ZIP) [file pone.0298601.s001.zip › Data/地级市Z_F工作报告.zip!/║■▒▒/╧╠─■╩╨/└┤╫╘╣1⁄2╓┌║┼í╛┬φ┐╦╩2╛▌═°í┐_2019.docx]

各位代表：

现在，我代表市人民政府向大会报告工作，请予审议，并请市政协各位委员和列席人员提出意见。

一、2018年工作回顾

2018年，市政府坚持以习近平新时代中国特色社会主义思想为指导，认真贯彻落实习近平总书记视察湖北重要讲话精神，全面贯彻落实党中央、国务院和省委、省政府的决策部署，在市委的坚强领导下，解放思想，担当作为，有效应对错综复杂的外部环境和国内经济下行压力，较好完成了市五届人大二次会议确定的目标任务，为改革开放40周年、咸宁建市20周年献上了一份厚礼。

——经济运行提速增效。预计地区生产总值增长8.5%左右；规模以上工业增加值增长8.5%；固定资产投资增长11.3%；一般公共预算收入可比增长12.1%；社会消费品零售总额增长11.9%；出口总额增长31.1%。税收占比提高6.3个百分点，居民收入与经济增长基本同步，节能减排可完成省定任务，经济运行呈现高开稳走、稳中向好、好中显优、持续发展的良好态势。

——特色产业不断壮大。做好现代农业、高新技术产业、全域旅游“三篇文章”。国家农业可持续发展试验示范区有力推进，农产品加工业总产值首次突破千亿元。高新技术产业增加值增长13.7%，占GDP比重10.7%。重点成长型产业集群数量跃居全省第一，世界500强企业投资咸宁达到38家，金盛兰集团入选湖北民营企业100强。旅游接待人数和旅游总收入均增长16%以上，三国赤壁古战场成功晋级国家5A级旅游景区。

——三大攻坚战成效显著。开展金融领域风险专项整治，商业银行不良贷款率降至1.86%。构建政府风险防控体系，政府债务风险总体可控。加大脱贫攻坚力度，8.46万人脱贫，48个村出列，通山、崇阳有望摘帽，3.39万人易地扶贫搬迁，贫困发生率降至1.06%。污染防治取得重大进展，空气质量在全国169个重点城市中排名第19位，荣膺全国水生态文明城市，入选全国黑臭水体治理示范市。

——改革创新亮点纷呈。系统推进98项重点改革项目，统筹推进经济、社会、生态等领域改革，形成了一批可复制可借鉴的重大成果。农村集体产权制度改革、市以下财政事权与支出责任划分改革、供销社合作发展基金试点、“三双”扶贫模式探索、农村物流三级配送体系建设等咸宁经验被推向全国。

——民生福祉持续改善。民生支出占一般公共预算支出77.7%。城镇新增就业4.91万人，城镇登记失业率2.68%。预计城乡居民人均可支配收入分别增长8%、8.5%。城乡低保标准分别提高9%、20%。退休职工基本养老金稳步增长，残疾人两项补贴全覆盖，特困供养制度全落实。教育、医疗、文化等民生事业全面发展。

一年来，我们主要做了以下工作：

（一）着力推进项目投资建设。把项目投资作为发展的主支撑，持续推进“三抓一优”。围绕“一座城”“一瓶水”“一部车”“一条路”“一机场”等“五个一”工程，积极谋划推进项目建设，累计入库项目1704个、总投资6693.5亿元。开展“一季一签约、一季一开工、一季一拉练、一季一排名、一季一通报”等活动，开工项目342个、总投资1419亿元，3个省领导督办项目、9个省级重点项目、9个10亿元以上新开工项目超额完成年度投资计划。把招商引资作为重中之重，举办专题招商活动40多场，新签约项目949个、总投资1809亿元，累计到位资金1122亿元，野马新能源汽车、安利XS运动饮料、华彬快消品等一批重点项目签约落户。建立“五位一体”考核机制，出台记功奖励办法，加大项目考核权重，形成了以发展论英雄的浓厚氛围。

（二）着力推进产业转型升级。坚定不移推进产业强市，加快构建现代化产业体系。三产结构比由15.6:48.4:36.0调整为14:49:37。持续实施“工业崛起”战略。规模以上工业企业新增115家，总数932家，预计利润增长14%，立邦涂料、维达力电子、华舟应急装备等一批投资10亿元以上的工业项目正式投产。创建省级隐形冠军示范企业2家、科技小巨人企业16家，5家企业上榜湖北民营企业制造业百强。加大企业投入力度，工业投资、技改投资分别增长20.9%、19.5%。积极发展现代农业。农业总产值329.49亿元，农业增加值增长3.4%。建成省级现代农业产业园3个，市级以上农业产业化龙头企业244家。推进农产品品牌建设，“三品一标”418个。成功举办中国咸宁首届农民丰收节，嘉鱼荣获中国鮰鱼之乡，赤壁入选中国茶叶百强县。加快发展第三产业。全年接待游客6700万人次，实现旅游总收入340亿元。松鼠部落、柃蜜小镇、果匠小镇、俄罗斯方块小镇等12个景区集中开园，第十届国际温泉文化旅游节成功举办。加快文化产业项目建设，预计文化产业增加值占GDP比重3％。大力发展电子商务，电商企业740家，淘系网商过万户。贺胜金融小镇落户基金公司246家，资金管理规模504亿元。开展消费促进活动，商品房销售面积和总额分别增长22.2%、52.4%。

（三）着力推动改革开放创新。以供给侧结构性改革为主线，不断激发经济社会发展活力。纵深推进“三去一降一补”，钢铁、煤矿去产能基本完成，商品房去化周期稳定在合理区间，降低企业成本10亿元。深入推进“放管服”改革，政务移动支付、不动产登记改革全省领先，成功入选国家政务服务和公共资源交易监管标准化试点市。统筹推进其他重点领域改革。河湖库长制实现全覆盖。盐业监管体制改革、国有林场改革圆满完成。财税、投资、信用体系等改革深入推进，监察体制改革、民兵军事训练改革、城管体制改革等形成经验，咸安农村福利院公建民营医养结合、嘉鱼公共文化服务体系建设、赤壁移民美丽家园建设、通山村级小微权力制度、通城殡葬改革、崇阳“联校网教”等试点工作走在全国全省前列。积极扩大对外开放。实现进出口总额5.56亿美元、实际利用外资4031万美元，同比分别增长10.1%、27%。国际友城数量增至19个。大力实施创新驱动战略。高新技术企业214家，授权专利1820件。建成国省级科技企业孵化器6家、省级众创空间9家。科技创新综合考评获省委、省政府通报表扬。加快金融创新步伐。推出“惠农贷”“畜牧贷”“小微快贷”等，获批省政府“金种子” “银种子”企业16家，挂牌新三板、四板企业265家。各项存贷款余额分别增长7.9%、13.4%，贷存比提高3.12个百分点。连续16年获评“湖北省金融信用市”。实施人才强市战略。表彰首批“南鄂英才”，举办“天津院士专家咸宁行”暨“站点引才”项目对接活动，“南鄂杯”全球高层次人才创新创业大赛影响力持续提升，“招硕引博”“双回双创”成果丰硕。增强市场活力，市场主体增至19.8万户，实有注册资本金3013.34亿元，分别增长15.16%、26.79%。驰名商标达到22件。获评中国质量魅力城市，入选全球可持续发展标准化城市联盟。

（四）着力推进生态文明建设。一体推进生态修复、环境保护和绿色发展，不断促进美丽咸宁建设。狠抓环保突出问题整改，中央、省级环保督察年度整改任务全面完成。大力推进长江大保护，完成37个非法码头整治，退腾复绿4035亩，恢复长江岸线6.6公里。实施“蓝天、碧水、净土”工程，PM2.5、PM10平均浓度分别下降21.3%、9.7%，城区空气质量优良率82.6%；8个县级以上城市集中式饮用水水源地、4个跨界断面水质全部达标；关闭禁养区内规模养殖场148家，恢复治理矿山地质环境3235亩。实施“五个三”重大生态工程，建成乡镇垃圾压缩中转站60个、污水处理厂48座，新建改造厕所86185座，精准灭荒完成面积全省第一，森林覆盖率50.44%，巩固提升农村安全饮水15.15万人。启动餐厨垃圾无害化处理、建筑垃圾资源再利用和垃圾强制分类工作。开展“一山两线”生态修复和人居环境综合整治。市城区拆围建绿，新建小游园，初步实现300米见绿、500米见园。新增省级生态乡镇7个、省级生态村54个、省级森林城镇3个、省级绿色示范乡村58个，崇阳、通山获批省级生态文明建设示范县。全面开展领导干部自然资源资产离任审计，长江大保护相关环境指标纳入政绩考核，建立生态保护补偿工作机制，生态文明体制进一步完善。

（五）着力推进城乡融合发展。加强基础设施和公共服务建设，实现城乡区域互促共进。强化规划引领。基本完成“多规合一”城乡总体规划，正式启动公园城市建设，北部空间长岛未来城、恒大健康谷等重大项目相继入驻，全市城镇化率53.7％。补齐基础设施短板。武深高速嘉鱼段全线通车，嘉鱼长江大桥、赤壁长江大桥建设进展顺利，107国道咸安赤壁绕城段、咸宁大道西延伸段开工建设。完成一二级公路建设204.35公里，提档升级农村公路1432.5公里，创建“四好农村路”600公里，农村公路生命安防工程四年任务两年完成。水利补短板争取投资11.9亿元，创历史新高。推进公共服务建设。市美术馆正式开馆，嘉鱼非遗馆、通山博物馆、通城文化馆投入使用。新增海绵城市规模2.3平方公里、城市地下综合管廊7.75公里。咸宁公交通过国家公共服务标准化试点验收。统筹推进“三带”发展。咸嘉生态文化城镇带提质增效，重点实施项目44个，总投资90亿元。幕阜山绿色产业带总体发展规划及四项政策相继出台，旅游公路主支线路段均已建成。编制完成《咸宁长江经济带生态保护和绿色发展重大项目规划》，与中国节能环保集团签订战略协议，启动沿江生态文明示范带建设。强力推进乡村振兴。熊家湾、石门村、茅井村等一大批美丽生态宜居乡村初见成效，23个田园综合体加快建设，10个村入选中国传统村落，通山宝石村入选中国历史文化名村，全市村级集体经济“空壳村”全部“销零”。

（六）着力推进民生事业发展。满足人民群众对美好生活向往，尽心尽力解决群众的急事难事。整合产业扶贫资金7.2亿元，落实产业扶贫项目571个。为贫困群众代缴城乡居民基本医疗保险和商业补充医疗保险1.7亿元，报销（补助）医疗费用4.78亿元。投入教育专项扶贫资金3.2亿元，“雨露计划”惠及贫困学子5439人。发放城乡低保、五保资金4.8亿元。改造棚户区9930套、农村危房6942户。新建、改扩建学校131所，招录农村义务教育教师749人，择校热、大班额等突出问题有所缓解。建立医疗服务价格动态调整机制，全面推行“两票制”、药品集中带量采购。启动“一村一名大学生村医”计划，全面落实家庭医生签约服务。出台全面二孩配套措施，二孩出生数占比51.33%。养老体系建设不断加强，每千名老人拥有养老床位数33.2张。弘扬社会主义核心价值观，“香城大舞台”被列为第四批国家公共文化服务体系示范项目。基层文化中心阵地建设实现全覆盖，通山“农村宗祠（祖祠）改建文化礼堂”工程“十个一”标准在全省推介。全国文明城市创建拉开序幕，创建工作初见成效。成功举办2018咸宁国际温泉马拉松赛、超百公里极限挑战赛、“探秘幕阜山”汽车旅游集结赛、首届咸宁龙舟公开赛。推进食品安全示范城市创建工作，完成“明厨亮灶”892家，“一书四员”制度得到上级肯定。安全生产总体稳定，水上交通安全生产四项指数连续22年保持为零。平安咸宁、法治咸宁建设深入推进，扫黑除恶创全省“九个第一” ，依法行政工作连续三年全省第一。稳妥处置涉稳事件，大力化解信访积案，办理法律援助案件3167件，人民调解工作走在全省前列。

（七）着力转变政府工作作风。深入学习习近平新时代中国特色社会主义思想和习近平总书记视察湖北重要讲话精神，坚决贯彻落实上级党委政府的决策部署。扎实开展支部主题党日活动，推进“两学一做”学习教育常态化制度化。严格落实全面从严治党主体责任，认真贯彻中央八项规定实施细则，深入推进政府系统党风廉政建设和反腐败斗争。全面推进阳光政务，电子政务外网实现市县乡村四级全覆盖。市领导联系代表委员、企业、重点项目和走访企业活动深入开展，全媒体问政、政风行风热线等政民互动渠道更加畅通。自觉接受人大法律监督和政协民主监督，办理人大代表建议116件、政协提案126件，办复率均为100%，满意率分别为99.13%和98.41%。加强与工会、共青团、妇联等群团组织的联系。

双拥创建、国防动员和后备力量建设进一步加强，中国海军咸宁舰正式入列。对口支援新疆、建始县工作深入推进。妇女儿童、青少年、关心下一代、红十字、老龄、残疾人等事业全面发展。对台、侨务、科普、保密、档案、方志、统计、气象、人防、能源、通讯、民族宗教、防震减灾、住房公积金、新闻出版、社科、文联、决策咨询等工作取得新成绩。

各位代表，2018年是改革开放40周年，也是咸宁建市20周年。40年来，特别是建市20年来，全市人民以厚德尚学、勇毅笃行的咸宁精神，攻坚克难，负重奋进，绘就了鄂南大地振兴崛起、化茧成蝶的壮丽画卷，谱写了香城泉都激情四射、放飞梦想的美好乐章。回顾历史，我们深深感到，所有成绩的取得，是以习近平同志为核心的党中央坚强领导的结果，是省委、省政府关心厚爱的结果，是市委统揽全局、协调各方的结果，是市人大、市政协支持监督的结果，是全市上下团结拼搏的结果。在此，我代表市政府，向全市广大干部群众，向人大代表、政协委员，向各民主党派、工商联、人民团体和社会各界人士，向驻地人民解放军、武警官兵和国家、省驻咸单位，向所有关心、支持、参与咸宁改革发展和现代化建设的海内外朋友们，表示崇高的敬意和衷心的感谢！

在看到成绩的同时，我们也清醒地认识到，发展不平衡不充分仍然是咸宁最大的实际。经济总量不大、结构不优、质量不高的问题尚未得到有效解决。传统产业占比较高，转型升级压力较大；新兴产业培育不够，新的经济增长点不多；产业竞争力不强，低端产业较多，产业链不完整；经济外向度不高，外贸支撑力有限；区域发展不平衡，民生领域还存在许多短板；少数干部能力不足、作风不实、担当不够等等。我们一定正视问题、直面挑战，坚决采取有力措施，切实加以解决。

二、2019年工作思路和主要任务

今年是新中国成立70周年，是全面建成小康社会的关键之年。党中央作出了我国发展仍处于并将长期处于重要战略机遇期的重大判断，省委谋划了“一芯、两带、三区”重大发展战略，市委确立了“133”区域和产业布局，这为我市发展提供了新动力、新机遇、新前景。同时，我国外部发展环境更加复杂严峻，国内经济运行稳中有变、变中有忧，长期积累的风险隐患有所暴露，这给我市发展带来了新压力、新挑战、新矛盾。在这场百年未有之大变局中，既蕴藏着重大机遇，又面临着重大挑战。但总体来讲，机遇大于挑战，时和势有利于我们加快发展，我市正处于发展的黄金机遇期。只要我们保持定力，抢抓机遇，迎难而上，完全有信心、有能力实现新的跨越，推动咸宁科学发展、高质量发展。

2019年经济社会发展工作思路：以习近平新时代中国特色社会主义思想和习近平总书记视察湖北重要讲话精神为指导，全面贯彻中央、省委经济工作会议精神，按照“五个坚持”和“六稳”总体要求，深入实施“创新驱动、绿色崛起”战略，坚决打好“三大攻坚战”，重点做好“现代农业、高新技术、全域旅游”三篇文章，大力促进“三带”协同发展，持续深化“三抓一优”，扎实推进“五个一”工程，全面做好稳增长、促改革、调结构、惠民生、防风险各项工作，加快建成全省特色产业转型发展增长极，建优建美长江流域公园城市，奋力谱写新时代咸宁高质量发展新篇章，为全面建成小康社会收官打下决定性基础，以优异成绩庆祝新中国成立70周年。

2019年经济社会发展预期目标：地区生产总值增长8%以上；规模以上工业增加值增长8%以上；固定资产投资增长11.5%；一般公共预算收入增长9%；社会消费品零售总额增长11.5%；进出口总额增长10%；居民消费价格涨幅控制在3.5%以内；城镇登记失业率控制在4.5%以内；居民收入增长与经济增长基本同步；生态环境进一步改善，完成省定节能减排目标。

实现上述目标，要着重抓好以下七个方面工作：

（一）稳定经济发展预期。以“六稳”为重点，有效应对经济下行压力，统筹发展举措，引导发展预期，提振发展信心，确保经济运行在合理区间。

扩大有效投资。发挥投资的关键性作用，深化“三抓一优”。开展“重大项目建设年”活动，加强“双十”项目调度，定期开展项目督查督办，推动项目早落地、早开工、早投产、早见效。抢抓国家、省政策机遇，确保有更多项目和资金纳入国家和省投资计划。围绕“五个一”工程，实施一批重大产业项目、基础设施项目、民生工程项目。集中力量抓好省领导重点督办项目、省级重点项目和10亿元以上新开工项目。建成嘉鱼长江大桥，加快赤壁长江大桥、咸九高速、107国道改扩建、咸宁大道西延伸段、航空护林站等重点项目建设。全力争取武咸城铁并入国家铁路网、武汉城市圈内环高速途经咸宁。加快咸宁机场、岳咸九铁路、铁公水多式联运项目、城区南外环高速、通修高速、仙崇高速等前期工作。加快实施黄盖湖防洪综合治理、斧头湖湖堤加固、陆水干流重点河段防洪治理等水利补短板项目，积极谋划鄂南防灾减灾水利工程。加强项目储备库、建设库、达效库建设，进一步提高入库项目转化率。统筹招商力量，创新招商方式，加大产业链招商力度，重点围绕主导产业、国内外龙头企业、行业隐形冠军、“独角兽”企业开展招商引资。筹备召开首届咸商大会，充分发挥咸商作用。全年引进国内外500强或行业龙头企业投资项目3个，投资额10亿元以上项目10个、亿元以上项目100个，招商到位资金1000亿元以上。

增强消费能力。发挥消费的基础性作用，积极扩大内需。大力推进农产品畅通工程，设立咸宁特色农产品展示展销窗口，全面开展农商互联、农餐对接、农超对接、农企对接活动。开展楚菜“一县一品”推介，推出一批咸宁特色菜肴。加快发展电子商务，创建市级电商示范基地2个，省级电商示范企业1家，市级电商示范企业5家。积极发展旅游地产、养老地产、特色民宿等，鼓励住房租赁消费，保持房地产持续健康发展。大力推进全域旅游，打造羊楼洞世界茶业第一古镇，提升九宫山旅游景区品牌，加快131军旅小镇、富水湖综合开发、陆水湖保护性开发、青山湖景区开发等项目建设，确保全年接待旅游人数7600万人次、旅游总收入390亿元。积极发展康养产业，推进梓山湖大健康产业示范区、康美小镇等建设。完善城乡物流快递体系，推进湘鄂赣边贸市场、咸安商贸物流区建设，加快华中国家绿色印刷包装物流产业园建设。积极营造良好消费环境，打造区域内强大消费市场。

推进开放合作。发挥外贸的支撑性作用，提升经济外向度。深入实施“走出去引进来”战略，有效应对中美贸易摩擦，帮助出口企业解难脱困。推进外贸“三项工程”，做强骨干企业、扶持成长企业、孵化开口企业。组织企业参加“走进非洲”“走进拉美”和“海上丝绸之路”活动，支持重点企业在海外建设营销网络、开展境外投资。积极打造电子电器、卫生材料、纺织服装、汽车零部件等优势出口产业，努力开辟农产品出口新领域。支持通城万雅进（出）口商贸交易中心建设。对接武汉自由贸易试验区，推动建立综合保税物流中心。对接粤港澳大湾区建设、长三角区域一体化等战略，积极开展经贸洽谈活动。主动融入“一带一路”，加强国际友城交流，办好2019“一带一路”国际茶产业论坛暨第五届国际茶业大会系列活动。深入推进与武汉经济开发区、东湖高新区合作，支持通城与湖南城陵矶新港区、岳阳三荷机场开展战略合作。

（二）推动经济高质量发展。坚持“质量第一、效益优先”，提升经济含金量、含新量、含绿量，实现经济高质量发展。

推进长江经济带绿色发展。继续实施长江经济带绿色发展十大战略举措，重点推进28个重大事项、88个重大项目建设。加快沿江产业转型升级，重点发展电子信息、高端装备、生物医药、新能源新材料等新兴产业，推进省级农产品优势区、综合物流园区、低碳经济示范区等项目建设。加强生态修复和环境保护，实施长江绿色生态廊道工程，完成长江沿岸造林绿化55000亩，全面启动幕阜山区森林质量精准提升示范工程，加快孝子山·双龙山生态修复及绿色发展示范区、桂花国家森林公园、向阳湖国家湿地公园等项目建设。大力实施循环发展引领行动、建筑垃圾资源化利用、固废综合利用、无害化处置工程等，推进国家生态文明示范城市、公园化特色小镇、生态宜居美丽乡村建设。加快绿色交易平台发展，持续推进碳排放权、排污权交易试点。

推动制造业高质量发展。积极推进“中国制造2025”试点示范城市建设，推动咸宁制造向高端提升，规上工业企业突破1000家。实施传统产业升级工程，滚动实施“万企万亿技改工程”，确保工业投资增长12%、技改投资增长15%以上。实施“隐形冠军”培育工程，培育国家级隐形冠军企业1家、省级隐形冠军企业2家、科技小巨人16家。实施新兴产业培育工程，推动12个重点产业集群发展壮大，培育五大千亿、五大百亿级的10个重点工业主导产业。实施“万企上云”工程，开展两化融合贯标试点，加快推动互联网、大数据、人工智能和制造业深度融合。推进军民融合产业发展，加快咸宁高新区军民融合产业园建设。

深入实施质量强市战略。实施品牌建设工程，引导更多的市场主体注册国家地理标志证明商标，积极争创全国和省级知名品牌示范区。开展“一县一品”质量提升行动，重点抓好人造板、天然石材、小麻花、防水卷材、涂附磨具、茶产业等6类品质提升。实施“荆楚优品”咸宁工程，积极申报湖北名牌、长江质量奖，继续开展市政府质量奖、咸宁名牌评选工作。实施标准化战略，推动“绿色咸宁”标准体系建设，打造咸宁特色标准化试点示范项目，开展城市可持续发展国际标准试点工作。全力推进全国质量强市示范城市创建活动，确保通过省级验收。

（三）坚决打好三大攻坚战。坚持尽锐出战、合力攻坚，打赢全面建成小康社会的关键之战。

防范化解重大风险。严厉打击非法集资、非法校园贷、电信网络诈骗、违规举债融资等行为，重点防控涉众型经济犯罪、互联网金融风险，加强风险监测、分析和预警，提升金融风险识别、防范和处置能力。强化全口径债务管理，落实限额及预算管理，加强政府投资项目管理，坚决遏制隐性债务增量，稳妥化解到期债务，确保不发生系统性风险。正确处理防范金融风险和解决融资难融资贵的关系。扎实做好社会稳定、公共安全、养老保险支付、房地产等领域风险防范化解工作。

打赢脱贫攻坚之战。下足绣花功夫，确保脱贫2.3万人，出列12个贫困村，通城县完成“摘帽”，实现区域性整体脱贫。加强产业指导、产销对接、金融支持，积极开发公益岗位、扶贫车间，帮助贫困户发展产业、实现就业。巩固脱贫成果，坚持脱贫不脱责任、脱贫不脱政策、脱贫不脱帮扶，加快推进贫困村十大提升工程。完善县级脱贫攻坚项目库，加大扶贫资金统筹力度，切实管好用好扶贫项目资金。开展扶贫扶智行动，加强政策舆论引导，激发贫困户的内生动力。全面压实扶贫主体责任，持续开展扶贫领域专项治理。

打好污染防治之战。继续实施“蓝天、碧水、净土”三大工程，开展柴油货车污染治理、建筑渣土扬尘污染整治、城市黑臭水体治理、斧头湖和陆水湖综合治理、农业农村污染治理攻坚战。深入实施“五个三”重大生态工程，新改建厕所6.9万座，精准灭荒16.9万亩，5个城区污水处理厂提标升级，57个乡镇生活污水处理项目全部投入运营。全面落实河湖库长制，大力开展清河行动。认真抓好中央、省级环保督察突出问题整改工作。继续强化非煤矿山整治，加快推进“矿山复绿”工程。开展“绿盾2019”自然保护区专项检查行动。创新和改进环境治理方式，加强对企业治污指导。落实领导干部自然资源资产离任审计、生态补偿、环保督察、环境司法责任等制度。

（四）大力推进改革创新。坚持向改革要红利，向创新要动力，向市场要活力，不断厚植发展新优势，培育经济增长新动能。

全面深化改革。深化供给侧结构性改革，全面落实巩固、增强、提升、畅通八字方针。巩固钢铁、煤炭去产能成效，确保不反弹。落实房地产调控政策，把控好房地产供给节奏，确保商品房去化周期控制在合理区间。优化降成本政策措施，全年为企业降低成本10亿元。实施补短板三年十大重点工程，建设“四好农村路”500公里。深入推进国有企业、商事制度、乡镇财政体制等重点领域改革攻坚。深化“互联网+政务服务”，推进“一网覆盖”“一次办好”。深化统计制度改革，扎实做好全国第四次经济普查工作。实施公平竞争审查制度。严格落实政府机构改革责任，确保如期完成改革任务。深化投融资模式改革，加快政府融资平台转型发展。

推进创新创业。实施高新技术产业发展工程，申报高新技术企业90家，高新技术企业总数达到240家，产业增加值占GDP比重达到11%以上。实施科技成果转化工程，加快建设咸宁网上技术交易平台，建设横沟科学城产学研一体化创新服务中心，新建省级技术创新平台2家，转化重大科技成果46项。实施科技企业创业与培育工程，推动科技企业孵化器提档升级，申报认定省级科技企业孵化器2家，新增科技创业企业（项目）100家（个）。加快推进智能机电国家级创新型产业集群建设，打造区域性智能机电产业中心。实施人才强市战略，深入推进南鄂英才计划，重点实施“站点引才”“咸商咸才回归”“南鄂校友资智回归”等计划。依托市青年企业中心和梦香城·人才创新创业超市，打造“政策+服务+平台+环境+创新”五位一体的大学生创新创业孵化体系。

激发市场活力。培育市场主体，推动“个转企”“小升规”，市场主体达到21万户以上。拓展市场空间，建立“走出去”公共服务信息平台，支持企业到市外开展并购等业务。优化市场环境。全面实施市场准入负面清单制度，落实普惠性减税政策，加强社会信用体系建设。简化企业办事流程，6月底前将企业开办时间压缩到3个工作日内，将项目建设平均审批时限压减至39个工作日内。大力支持民营企业发展。建立健全领导联系民营企业制度、民营企业家联席会议制度。深入开展银企对接，推进小微和民营企业金融服务示范区建设，确保贷款企业和A级以上信用企业数量年增长10%。积极支持企业上市。鼓励民营企业技术创新。搭建民营企业服务平台，构建亲清新型政商关系。

（五）全面实施乡村振兴战略。坚持“20字方针”，实施三年行动计划，加快“五个振兴”，奋力谱写乡村振兴新篇章。

发展现代农业。深入推进优质粮食工程建设，切实保障粮食安全。推进国家农业可持续发展试验示范区、农业绿色发展试点先行区和杨畈农高区建设，做强千亿农产品加工业，新增省级以上农业产业化龙头企业12家。调整优化农业产业结构，创建3个省级特色农产品优势区，发展观光农业，推进白水畈田园综合体等建设，办好湖北·咸宁首届油菜花节。实施品牌强农战略，加强“西凉湖桂花鱼”“咸安桂花”“嘉鱼虾稻香米”“赤壁青砖茶”“通山枇杷”“崇阳雷竹”“通城油茶”等品牌建设，新认证“三品一标”20个。大力推进农业机械化，提高农业生产能力。

深化农村改革。整市推进农村集体产权制度改革试点工作，全面完成“两清”工作，发展农村集体经济组织。深化农村土地制度改革，盘活农民闲置宅基地和闲置农房，积极探索农村集体用地模式和农村土地抵押贷款办法。组建农村综合产权交易中心，推进农村产权交易。完善农村双层经营体制，新增农民专业合作社300家、家庭农场200家。深入实施“三乡”工程，引导“三乡”主体与村集体和农民开展股份、租赁、流转等合作，充分激活乡村振兴主体力量。

建设美丽乡村。全面实施“百村引领、千村共进”美丽乡村建设行动，高标准建成60个美丽乡村，新启动65个美丽乡村建设，重点抓好30个市级以上美丽乡村建设。深入开展农村人居环境“三清三治”三年行动计划，强力推进农村环境综合治理“六大专项行动”。加强基层文化中心阵地和法治文化示范点建设，大力实施“祠堂改建文化礼堂”工程。加强名镇名村、古桥古树、民俗文化的传承保护和适度开发。

（六）大力促进区域协调发展。落实“133”区域和产业布局，优化城市空间布局，提升城市内涵品质，促进城乡融合发展。

优化空间布局。按照“133”发展战略，调整完善城乡建设规划及相关专项规划，启动市域国土空间规划编制。做优主城区。推进咸宁宜居主城、横沟科学城、梓山湖健康新区、官埠生态新区、凤凰新区等 “一主四辅”建设，打造对接大武汉的桥头堡。提升县城区。支持县市区按照各自定位，突出主导产业，实现错位发展，夯实县域经济实力，形成全省特色产业增长点。打造特色小镇。积极推进沿江沿边沿路城镇规划建设，强化产业支撑，提升特色内涵，重点建设20个特色小镇。建设宜居乡村。加强农村建房管理和规划建设，以18个省级试点村为重点，打造具有鄂南特色的乡村模式。

提升城市品质。实施公园城市建设三年行动计划，推进主城区公园城市“十大工程”，启动县市公园城市建设。统筹推进淦河流域综合整治工程，切实解决城市内涝问题。稳步实施老城区疏解工程，加大老旧小区、背街小巷改造力度。深入推进“城市双修”，实施金桂路综合整治工程，加快小游园、小绿地建设，加强城区郊区山体保护。推进五彩咸宁建设，实施桂乡大道、银泉大道和咸宁大道彩化工程。加快建设市文化中心、金融信息港，建成市档案馆。实施交通品质提升工程，打通城市微循环，推进停车场、充电桩建设。加快智慧城市建设，支持5G建设，推进综合执法和市容市貌整治，提升城市治理精细化水平。推广绿色建筑，推进节能减排，倡导低碳生活。

推动三带发展。加快建设咸嘉生态文化城镇带，重点实施35个项目，大力推进高新区新能源乘用车、大洲湖生态建设示范区、向阳湖文化名人核心区、官桥新材料小镇、华夏幸福产业新城、恋江湖生态文化旅游区等项目建设。加快建设幕阜山绿色产业带，重点实施90个项目，全面完成旅游公路主支线标准化建设，积极打造通城、崇阳、通山境内三个重点示范段。大力建设沿江生态文明示范带，加强与中国节能环保集团合作，启动长江干堤绿化行动及“智慧长江”生态环境监管体系，推进咸宁市静脉产业园、嘉鱼滨江生态环境提升、赤壁凤凰山生态修复及绿色城市开发等示范项目。

（七）切实保障和改善民生。坚持以人民为中心，倾注更大力量补齐民生短板，使人民群众有更多的获得感、幸福感、安全感。

加强民生保障。加大民生投入，确保民生支出占一般公共预算支出比例76%以上。实施就业提升计划，鼓励大学生留咸回咸就业，推进农民工等人员返乡创业，全年新增城镇就业3.7万人。加快完善多层次社会保障体系，推进建筑领域农民工参加工伤保险。实施统一的城乡居民基本医疗保险制度和大病保险制度。建立城乡居民基础养老金正常调整机制。扎实做好困难群众帮扶救助工作，推进农村低保和扶贫政策有效衔接。健全解决农民工工资拖欠问题的长效机制。加强公办养老机构建设，建立健全智慧居家养老服务体系。扎实推进棚户区和农村危房改造。加快创建国家食品安全示范城市。加快新型城镇化进程，城镇化率达到54.9％。

发展社会事业。把教育摆在优先发展位置，继续实施主城区义务教育三年行动计划，加大全市新建改建学校力度，推进全市义务教育均衡发展，逐步化解“择校热”“大班额”等难题。启动实施高中阶段教育普及攻坚计划。推进学前教育和职业教育。支持湖北科技学院“双一流”建设，支持首义学院嘉鱼校区建设，支持咸宁职业技术学院创建国家优质高职。深化医药卫生体制改革，推进国家医养结合试点工作，加快医疗共同体建设，落实“一村一名大学生村医”计划，促进中医药振兴，全面推进健康咸宁建设。大力发展慈善和红十字事业。推进计划生育服务转型，完善全面二孩配套政策，关爱留守儿童。深入推进全国文明城市创建活动，加快“五城同创”，大力弘扬新时代咸宁精神。实施文化惠民工程，推进“香城书房”布局建设，加强古名居保护，抓好万里茶道申遗工作。继续开展咸宁国际温泉马拉松赛、咸宁龙舟公开赛等活动。加强公共服务能力建设，着力解决老百姓出行难、停车难、买菜难、如厕难等生活问题。

创新社会治理。加强基层民主法治建设，构建多元化处置机制，依法化解信访积案和历史遗留问题。深入开展扫黑除恶专项斗争，大力推进“雷霆行动”，扎实做好“雪亮工程”，持续开展禁黄、禁赌、禁毒专项行动和缉枪治爆、危险化学品、物流寄递专项整治。强化食品药品监管，实施餐饮品质提升工程，确保“舌尖上的安全”。开展安全生产打非治违专项行动，严格执行禁鞭条例，坚决防范遏制重特大事故发生。

继续做好国防动员、退役军人事务、民族宗教等工作，加强决策咨询和智库建设，支持群团组织发挥更大作用。

三、全面加强政府自身建设

各位代表，时代是出卷人，我们是答卷人，人民是阅卷人。我们要不忘初心，牢记使命，不懈奋斗，不断提升工作能力，建设人民满意政府。

（一）政治坚定，牢记使命。树牢“四个意识”，坚定“四个自信”，坚决做到“两个维护”，把旗帜鲜明讲政治贯穿到政府工作全方位、全过程。坚决贯彻落实中央、省委和市委的决策部署，结合实际创造性开展工作。学好用好习近平新时代中国特色社会主义思想这个金钥匙，提高谋划工作的能力、履职尽责的能力、贯彻落实的能力，增强做好经济工作的本领。

（二）依法行政，接受监督。统筹推进地方行政立法、行政执法、法律事务管理和普法宣传，落实宪法宣誓制度，推进公正文明执法，加快法治政府建设。加强合法性审查和风险评估，健全政府科学决策、民主决策、依法决策机制。自觉接受市人大及其常委会法律监督和工作监督、人民政协民主监督，主动接受社会舆论和人民群众监督。认真听取人大代表、政协委员意见建议，听取民主党派、工商联、无党派人士和人民团体意见。

（三）实干为先，敢于担当。加强政府绩效管理，全面提高政府执行力和效能。实打实、硬碰硬推进工作，少一些花拳绣腿，多一些真刀真枪。出台高质量发展评价体系，完善激励容错机制，使干部敢扛事、愿做事、能干事。优化政务环境，使咸宁真正成为亲和之地、创业之乡、致富之源。

（四）心系群众，执政为民。深入基层、深入群众，倾听群众呼声。以人民满意为最高工作标准，抓住老百姓最急最忧最怨的问题，解决好群众最关心最直接最现实的利益问题，为人民群众办实事、解难题，让民生幸福的底色更厚重，让共享发展的主题更温暖。

（五）从严治政，永葆清廉。严守政治纪律、政治规矩，认真履行管党治党主体责任，加强廉洁自律建设。紧盯不敬畏、不在乎、喊口号、装样子的问题，坚决破除形式主义和官僚主义。强化审计、财务监督，重点对公共资源配置、工程领域招投标、资金资源密集区域、扶贫资金使用等进行监管，严惩群众身边不正之风和腐败问题。知行合一，勤勉尽责，干净做事，决不辜负人民的期望。

各位代表，追梦需要激情和理想，圆梦需要奋斗和奉献。让我们紧密团结在以习近平同志为核心的党中央周围，在省委、省政府和市委的坚强领导下，凝神聚力、锐意进取、智圆行简、实心实政，为加快建成全省特色产业转型发展增长极，建设社会主义现代化强市而努力奋斗！
